# Kinetic investigations of sulfite addition to flavanols

**DOI:** 10.1038/s41598-020-69483-0

**Published:** 2020-07-30

**Authors:** Federico Bonaldo, Graziano Guella, Fulvio Mattivi, Daniele Catorci, Panagiotis Arapitsas

**Affiliations:** 1 Department of Food Quality and Nutrition, Research and Innovation Centre, Fondazione Edmund Mach (FEM), San Michele all’Adige, Italy; 2 Bioorganic Chemistry Laboratory, Department of Physics, University of Trento, Trento, Italy

**Keywords:** Organic chemistry, Chemical physics, Analytical chemistry

## Abstract

Flavanols are an important class of natural products occurring in almost all plants, fruits and vegetables; they have a great influence on wine ageing potential, astringency, colour stability and biological activities. In wine, flavanols react with sulfur dioxide ($\hbox {SO}_2$), the most widely used preservative in oenology, leading to sulfonated products. Here we report a kinetic investigation, through LC-MS quantitative measurements carried out at different pH (3 and 4) and temperature values (23, 30, 40, 50 and $60\, ^\circ \hbox {C}$), of the reaction products obtained by $\hbox {SO}_2$ addition to both monomeric (epicatechin and catechin) and dimeric flavanols (procyanidin B2 and procyanidin B3). The results proved that: (a) the major sulfonation route that leads quickly and in good yields to monomeric 4$\beta$-sulfonated derivatives passes through the acid-catalysed depolymerisation of proanthocyanidins; (b) monomeric flavanols lead to the same 4$\beta$-sulfonated products, although in a considerably slower manner, and also to other sulfonated regioisomers; (c) the kinetic data in our hands, in particular the temperature dependence of the observed rates, suggest the involvement of two completely different reaction mechanisms for the $\hbox {SO}_2$ addition to dimeric and monomeric flavanol substrates; (d) direct sulfonation of epicatechin is slightly faster than that of catechin.

## Introduction

**Figure 1:**
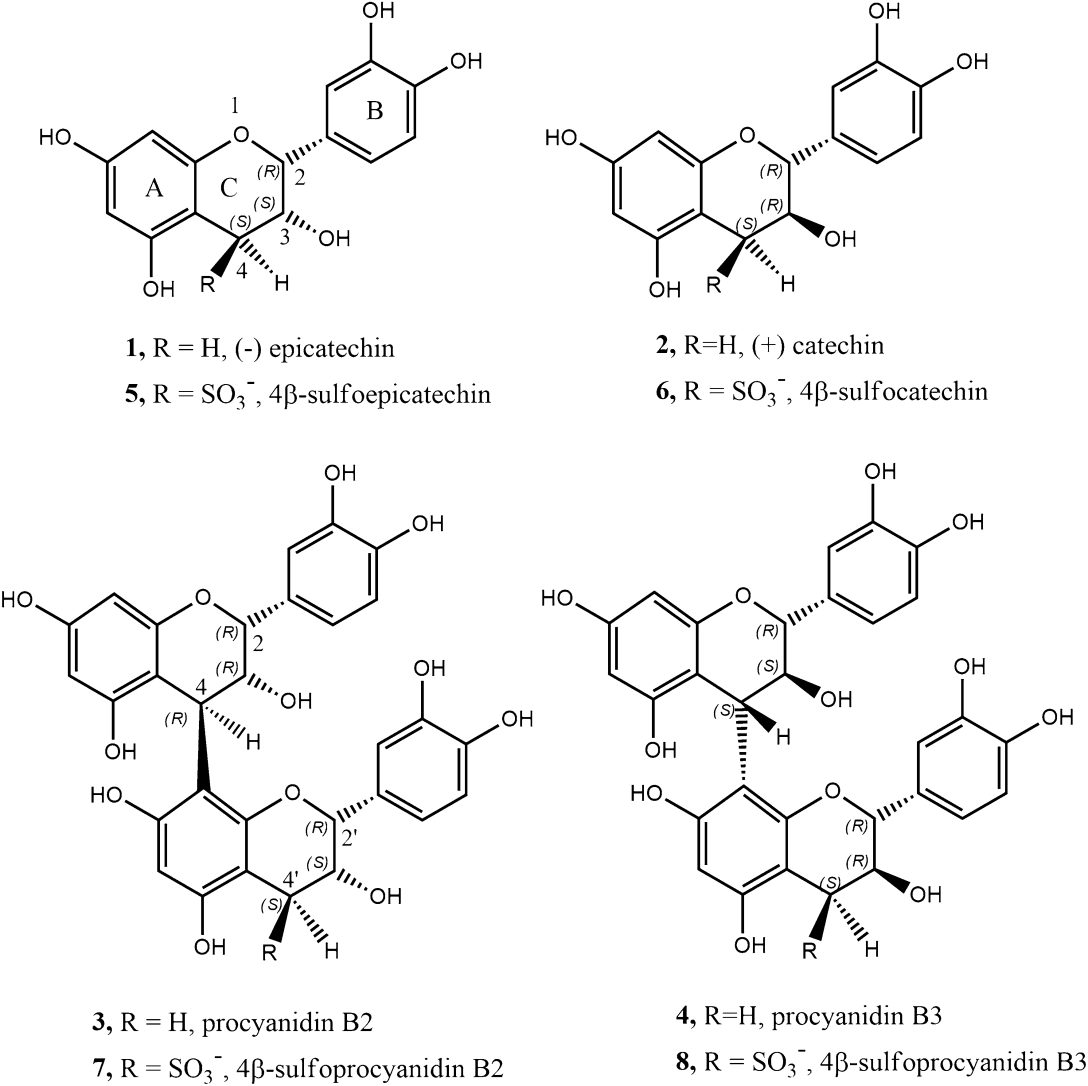
Structure of monomeric flavanols **1**-**2**, dimeric flavanols **3**–**4** and their corresponding 4-sulfo analogues **5**–**6** and **7**–**8**.

Flavanols are among the most important groups of secondary metabolites, due to their ubiquity, biological activities, nutritional value and food quality impact. They include the monomeric epicatechin (**1**), catechin (**2**), gallocatechin, epigallocatechin gallate, catechin gallate, oligomeric procyanidins (e.g. procyanidin B2 (**3**) and procyanidin B3 (**4**)), prodelphinidins, and polymeric proanthocyanins (Fig. 1). Condensed tannins are defined as oligomeric/polymeric flavanols with mass higher than 500 Dalton^1^. This group of secondary metabolites is found in most of the plants, fruits, vegetables and beverages, and so every day we intake huge amounts of them by consuming wine, tea, cocoa, coffee, chocolate, berries, apples, nuts, dry fruits, mint, basil, etc.^2–4^. The first scientific works about tannins were focused on their utility in the leather industry^1,5,6^, but today they are known and worldwide studied for their role in (a) human health^3,7,8^, (b) plant physiology and defence^2,9^ and (c) contribution to sensorial character of food, due to their astringent and bitter taste, or their ability to stabilise wine red colour^10–14^. Their properties depend on their chemical structure, such as degree of polymerisation, B-ring hydroxylation and C-ring configuration^15–20^. Centuries ago, the addition of $\hbox {SO}_2$ to a tannin extract was a key process in order to obtain better quality material for leather manufacturing^21^. Today, $\hbox {SO}_2$ (E220) addition is permitted and regularly used in a wide range of food including wine, dried fruits and meat products, because of its preservative effects^22^. However, $\hbox {SO}_2$ and sulfites are among the food allergens and the added doses are subject to legal limits^23,24^.

**Figure 2:**
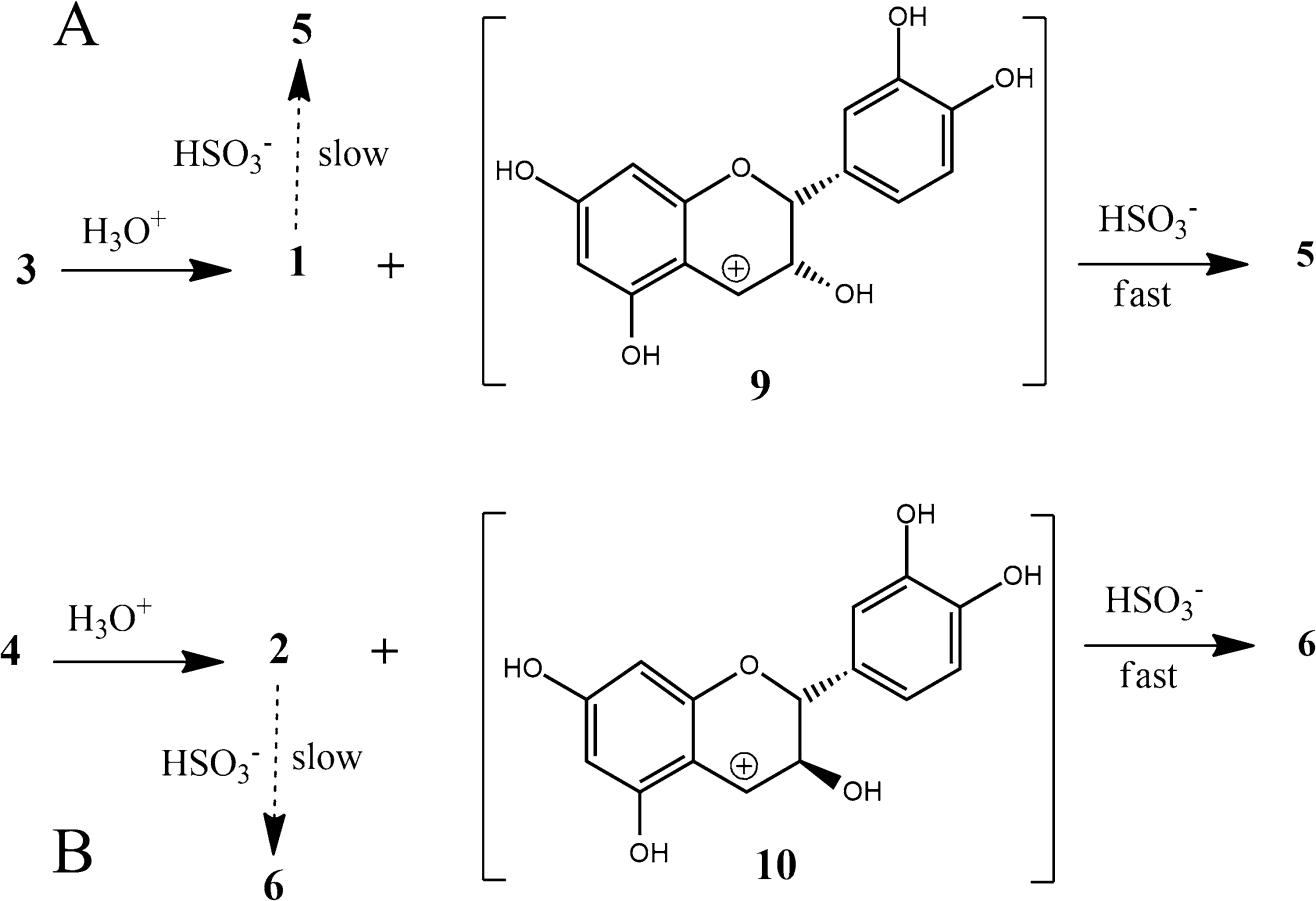
(**A**) Proposed hypothetical mechanism for the production of 4$\beta$-sulfonate **5** from interflavanic bond cleavage of procyanidin B2 (**3**); (**B**) production of 4$\beta$-sulfonate **6** from interflavanic bond cleavage of procyanidin B3 (**4**). A different mechanism is required for the slower conversions **1** $\rightarrow$ **5** and **2** $\rightarrow$ **6**.

A few years ago the monomeric epicatechin 4$\beta$-sulfonated (**5**) and the dimeric procyanidin B2 4$\beta$-sulfonated (**7**) were detected and quantified in wine^22,25,26^, clearly deriving from the addition of $\hbox {SO}_2$ to flavanols. Together with sulfonated epigallocatechin, these three are the only sulfonated compounds that have been isolated and fully structurally characterised^26–28^, and they all belong to the *epi*- (2,3-*cis*) conformation. It was found that this sulfonation reaction in wine was favoured by ageing and higher than optimal storage temperature of the wine^22,25^. Indeed, in aged wines the concentration of sulfonated flavanols was found to be much higher than in just-produced wines. The mechanism of their formation is still uncertain, but one reasonable hypothesis involves the acid-catalysed interflavanic bond cleavage of the polymeric tannins and/or oligomeric procyanidins that should then deliver these sulfonated derivatives (Fig. 2) as first and essential step^27^. Acid-catalysed interflavanic bond cleavage is a naturally occurring process in wine tannins, since it is favoured by the wine acidic pH^29–31^. Phloroglucinolysis and thiolysis protocols take advantage of this interflavanic bond cleavage in order to quantify extension and terminal units, as well as to calculate the mean degree of polymerisation of condensed tannins^32–34^. To check if the sulfonation process in wine starts with the acid-catalysed cleavage of the polymeric proanthocyanidins, producing the intermediate electrophilic carbocation species at C(4) (**9** starting from **3** or **10** starting from **4** in Fig. 2), it is necessary to know what the true products of the reaction and the main kinetic parameters (reaction order, rate constant, activation energy) are; these information are largely lacking in the current literature^26,27^.

**Figure 3:**
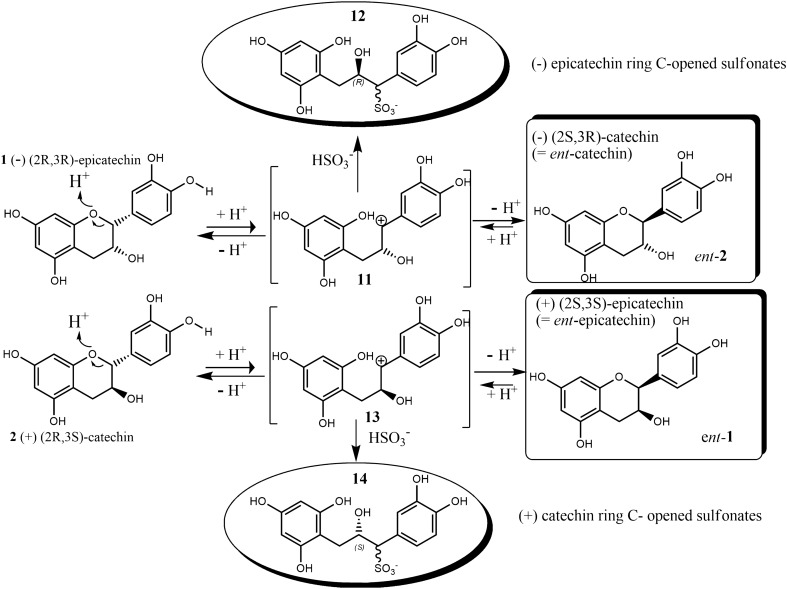
Top: acid-induced C(2) epimerisation of (−) epicatechin (**1**) leading to *ent*-**2** and/or, in presence of hydrogen sulfite, epicatechin ring-C opened sulfonates (**12**); Bottom: acid-induced C(2) epimerisation of ($+$) catechin (**2**) leading to *ent*-**1** and/or, in presence of hydrogen sulfite, catechin ring-C opened sulfonates (**14**).

Another essential chemical feature of flavanol substrates, such as **1** and **2**, which has been largely overlooked, is the stereochemical consequence imposed by the thermal (pH-dependent) isomerisation processes, triggered by ring-C opening/reclosure after breakage of the O–C(2) bond and causing epimerisation at the chiral centre C(2). In fact, (−)(2R,3R) epicatechin (**1**) can be thermally converted (at least partially) through this process into (2S,3R)(−) catechin (*ent*-**2**) whilst, in turn, ($+$)(2R,3S) catechin (**2**) can be converted into (2S,3S)($+$) epicatechin (*ent*-**1**) (Fig. 3)^35–37^.

Whether this process occurs in a similar timescale as the sulfonation process or whether the ring opening occurs by acid induced processes, it is clear that sulfonation of epicatechin should lead to epicatechin- and *ent*-catechin-sulfonated derivatives and similarly, sulfonation of catechin should lead to both catechin- and *ent*-epicatechin-sulfonated derivatives. Moreover, the ring-C opened carbocationic intermediates (**11** and **13** in Fig. 3) involved in the C(2) epimerisation are not only able to produce the corresponding epimer, but also to be captured by any nucleophile (e.g. $\hbox {SO}_2$) affording a 1-sulfo-2-hydroxy propyl moiety linked to ring A and B^26,38^. Actually, sulfonation processes carried out at high temperatures (favouring epimerisation) lead to ring-C opened C(1)-sulfonated end products (top and bottom products in Fig. 3). Thus, regio- and stereochemistry must be carefully considered in any kinetic investigation of flavanols, because they allow to obtain relevant information on the mechanistic details of the process. To our knowledge, no epicatechin or catechin enantiomers (*ent*-**1** and *ent*-**2**) were found in wine, but their detection in wine analysis is difficult in the absence of chiroptical tools.

Further complexity in these kinetic studies is given by the new chiral center produced at C(4) after sulfonation. Since the nucleophilic $\hbox {HSO}_3^-$ species (or the sulfur-centred radical anion $\hbox {SO}_3^{\cdot -}$) can add to the same or the opposite side of HO-C(3), two different diasteroisomeric products should be obtained on addition to epicatechin-based flavanols and also two diasteroisomeric products from catechin-based ones. Recently, we reported that the stereospecificity of this attack on epicatechin-based flavanols^26^ causes the addition of the sulfo-group at C(4) in a *trans* stereochemical relationship ($\beta$ oriented in the mean plane of ring C) to the $\alpha$ oriented HO–C(3), thus leading to (2R,3S,4R)-4-sulfoepicatechin (**5**) or 4$\beta$-sulfoprocyanidin B2 (**7**).

Differently from 4-sulfonated epicatechin flavanols, which were only recently investigated^22,28^, no previous studies have been reported on the stability and the relative stereochemistry of 4-sulfonated catechin analogues. Thus, we have also focused our attention on the kinetics of formation of the sulfo-derivatives of catechin (**2**) and procyanidin B3 (**4**). More generally, the aim of this work was to carefully evaluate the kinetic parameters of the sulfonation processes of monomeric (**1** and **2**) and dimeric flavanols (**3** and **4**) at different pH values, paying attention to the above outlined stereochemical aspects and side reactions prone to work in these conditions.

Indeed, we now have strong evidences of the production (although at lower specific rate) of both **5** and **6** starting from their respective monomers **1** and **2**, besides the already known monomeric sulfonation starting from substrate **3** and the newly discovered starting from **4** (Fig. 2). This outcome clearly indicates that other pathways, besides the quenching of the electrophilic carbocation intermediates **9** or **10** by the nucleophilic $\hbox {SO}_2$ species, must be considered.

## Results and discussion

### Kinetics of sulfonation processes of monomeric flavanols **1** and **2**

Epicatechin (**1**) was found to produce mainly epicatechin $4\beta$-sulfonate (**5**) (Fig. 2A) but not sulfonated open ring-C forms (Fig. 3). Actually, the extracted ESI(−) mass chromatogram of the anion at m/z 369.03 (expected for the molecular formula of a monomeric sulfoderivative $\hbox {C}_{15}\hbox {H}_{13}\hbox {O}_9\hbox {S}^-$) showed the presence of two minor isobaric species, besides the major **5**, at different retention times (Supplementary Fig. S1 online). MS/MS measurements on this isobaric ions allowed us to propose the presence of sulfonated products at ring B ($\sim 15\%$ of the major **5** at pH 3); worth of note, the presence of ring-B sulfonated products becomes comparable to the major **5** at pH 4. We describe here only the kinetics of formation of **5**, because the formation of the other isomers was so slow to be difficult to detect at lower temperature and in any case difficult to quantitate also at higher temperature.

**Figure 4:**
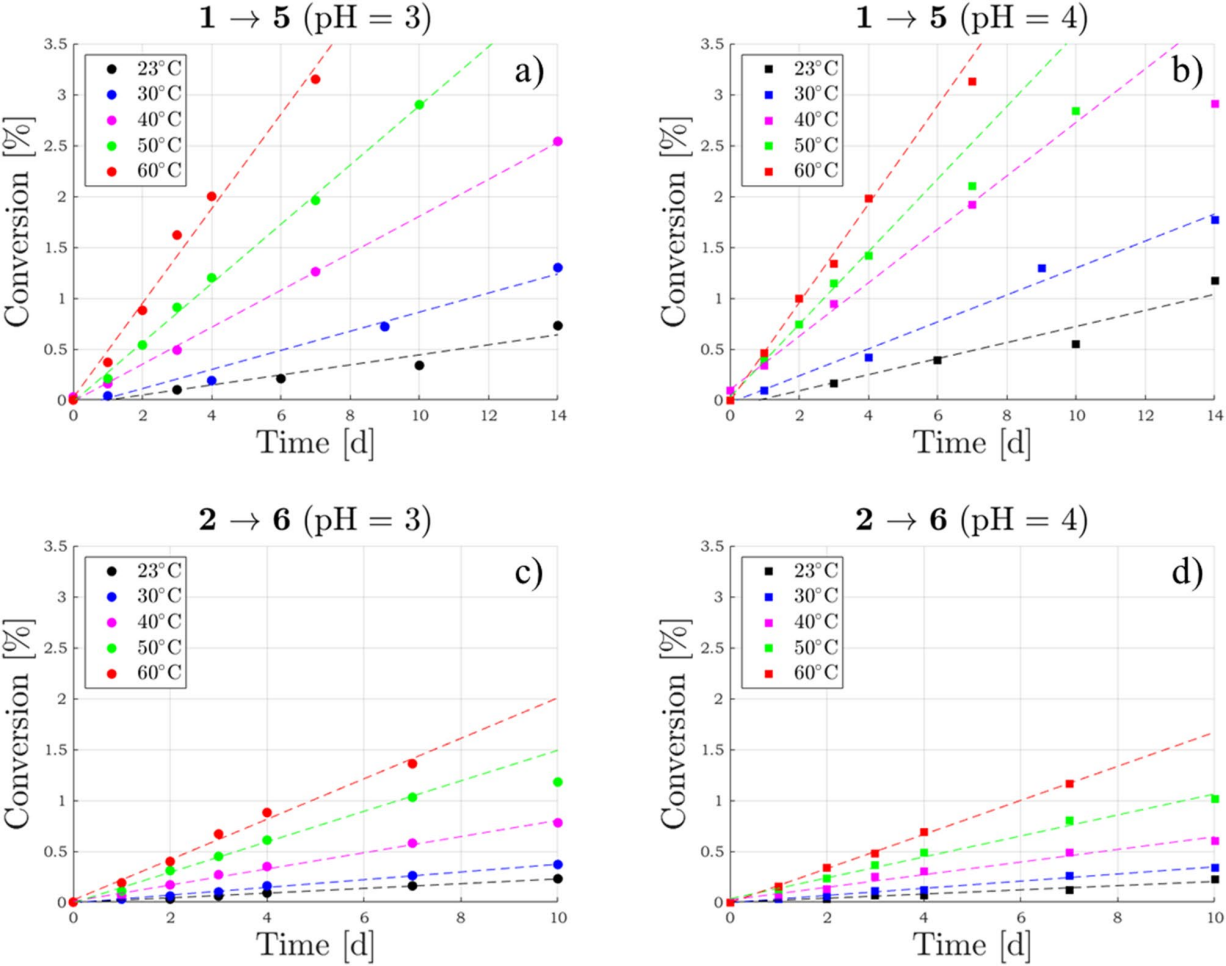
Comparison of the different temperature kinetics of epicatechin 4$\beta$-sulfonate (**5**) and catechin 4$\beta$-sulfonate (**6**) formation starting, respectively, from epicatechin (**1**) (**a, b**) and catechin (**2**) (**c, d**) at pH 3 and 4. R$^2$ coefficients can be found in Supplementary Table S3 online.

The time-dependent conversion of product **5** was followed for several days both in solution buffered at pH 3 (Fig. 4a) and at pH 4 (Fig. 4b) by ESI/MS quantitative measurements. The reaction progress was monitored at five different temperatures within the range $23\hbox {-}60\, ^\circ \hbox {C}$ in order to evaluate the corresponding activation energy (Supplementary Fig. S2 online). Measurements at temperatures outside this range were avoided because (i) below $23\, ^\circ \hbox {C}$ the progress of the reaction was found to be so low that products were undetectable and (ii) above $60\, ^\circ \hbox {C}$ thermally induced epimerisation at C(2) become quite significant^26,39^, thus making data analysis too complex.

The rate constant of the process **1** $\rightarrow$ **5**, at fixed pH and temperature values, was evaluated on the basis of two main considerations/assumptions:

- The sulfonation of **1** was considered a pseudo first order process since $\hbox {HSO}_3^-$ was used in large molar excess (50:1) with respect to **1** in all our experiments; this leads to the working equation $[\mathbf{1} ]_t = [\mathbf{1} ]_0 (exp\{-k_{obs} * t\})$; but, being at all times $[\mathbf{1} ]_0 = [\mathbf{1} ]_t + [\mathbf{5} ]_t$ it follows $[\mathbf{5} ]_t = [\mathbf{1} ]_0 (1-exp\{-k_{obs} * t\})$ where $k_{obs} = k*[$HSO$_3^-]_0$.
- Due to the very low conversion of **1** to **5** ($< 3\%$ even at $60\, ^\circ \hbox {C}$ after several days), i.e. $k_{obs} * t \ll 1$, the time-dependence of the formation of **5** can be further simplified to $[\mathbf{5} ]_t = [\mathbf{1} ]_0 * k_{obs} * t$; thus the overall process becomes zero order in [**1**]$_t$ giving a linear time-dependent relationship and allowing to define the simple working equation: $\% conversion = 100 * [\mathbf{5} ]_t / [\mathbf{1} ]_0 = k_{obs} * t$.

Basically, $k_{obs}$ is expressed in SI time units [s$^{-1}$] but here we used $k_{obs}$ [day$^{-1}$] $= 86400 * k_{obs}$ [s$^{-1}$]. Of course, k$_{obs}$ is only a phenomenological kinetic parameter giving a quantitative measure of the timescale of these processes, but cannot be used to derive mechanistic considerations. However, it is very useful to have an estimate of the fate of flavanols and their oligomeric analogues (procyanidins, tannins, etc.) in the usual wine storage temperature/pH conditions. The values of these rate constants at different pH/temperature (and their errors estimated by linear regression parameter $\sigma$) of the process **1** $\rightarrow$ **5** were evaluated using the above mentioned equation and are reported in Table 1, together with the corresponding activation barriers (E$_A$ / A from ln(*k*$_{obs}$)/T$^{-1}$ in the Arrhenius plot or $\Delta \hbox {H}^{\ddagger }$ / $\Delta$S$^{\ddagger }$ from ln(*k*$_{obs}$/T)/T$^{-1}$ in the Eyring plot).

**Table 1:** Observed rate constants and kinetic parameters of the processes **1** $$\rightarrow$$ **5** and **2** $$\rightarrow$$ **6** at different pH; $$^{a)}$$ values calculated at $$30\, ^\circ \hbox {C}$$.

Process	T ($^\circ \hbox {C}$)	$k_{obs}$ (day$^{-1}$)	E$_A$ (kJ/mol)	$\Delta H^\ddagger$ (kJ/mol)	${}^{\mathrm{a}} \Delta {S}^{\ddagger}$ [kJ/(mol K)]	${}^{\mathrm{a}} \Delta {G}^{\ddagger}$ (kJ/mol)
**1** $\rightarrow$ **5** (pH = 3)	23	$(4.9 \pm 0.3) \times 10^{-4}$	51.1 ± 3.7	48.5 ± 3.7	$-0.238 \pm 0.012$	120.8 ± 5.2
	30	$(8.9 \pm 0.4) \times 10^{-4}$				
	40	$(18.0 \pm 0.3) \times 10^{-4}$				
	50	$(29.1 \pm 0.5) \times 10^{-4}$				
	60	$(50.5 \pm 2.7) \times 10^{-4}$				
**1** $\rightarrow$ **5**(pH = 4)	23	$(7.4 \pm 0.5) \times 10^{-4}$	41.7 ± 5.0	39.1 ± 5.1	$-0.266 \pm 0.016$	119.9 ± 7.1
	30	$(13.1 \pm 0.7) \times 10^{-4}$				
	40	$(21.9 \pm 1.9) \times 10^{-4}$				
	50	$(36.9 \pm 1.0) \times 10^{-4}$				
	60	$(48.4 \pm 1.6) \times 10^{-4}$				
**2** $\rightarrow$ **6** (pH = 3)	23	$(2.2 \pm 0.1) \times 10^{-4}$	54.0 ± 3.7	51.4 ± 3.7	$-0.236 \pm 0.012$	122.8 ± 5.2
	30	$(3.7 \pm 0.1) \times 10^{-4}$				
	40	$(8.2 \pm 0.2) \times 10^{-4}$				
	50	$(15.6 \pm 0.4) \times 10^{-4}$				
	60	$(24.2 \pm 0.4) \times 10^{-4}$				
**2** $\rightarrow$ **6** (pH = 4)	23	$(2.0 \pm 0.1) \times 10^{-4}$	49.7 ± 5.1	47.1 ± 5.2	$-0.250 \pm 0.017$	123.0 ± 7.2
	30	$(3.5 \pm 0.2) \times 10^{-4}$				
	40	$(7.3 \pm 0.3) \times 10^{-4}$				
	50	$(12.8 \pm 0.1) \times 10^{-4}$				
	60	$(18.3 \pm 0.4) \times 10^{-4}$				

A few remarks summarising the kinetic data for the process **1** $\rightarrow$ **5** must be underlined. First of all, although we are dealing with a quite slow process, the $\%$ conversion increases by one magnitude order from $23\, ^\circ \hbox {C}$ (0.05%/day) to $60\, ^\circ \hbox {C}$ (0.50%/day). Consequently, if a wine contained initially only monomeric form of epicatechin **1**, at the usual non-optimal wine storage temperatures ($25\hbox {-}30\, ^\circ \hbox {C}$) about 30%/year of the latter might be converted into its sulfonated derivative **5**, thus a significant conversion (more than 50%) should be observed in 2–3 years. This is probably an underestimate, since the hydrogen sulfite is able to give other monosulfonated products, as aforementioned. Second, the observed rate constants do not increase significantly ($< 3\%$) at pH 4 with respect to pH 3, thus indicating their substantial independence from the concentration of H$_3$O$^+$. Curiously, the activation energy of this process was found to be significantly higher at pH 3 (E$_A$ = 51.1 ± 3.7 kJ/mol or $\Delta \hbox {H}^{\ddagger }$ = 48.5 ± 3.7 kJ/mol) than at pH 4 (E$_A$ = 41.7 ± 5.0 kJ/mol or $\Delta \hbox {H}^{\ddagger }$ = 39.1 ± 5.1 kJ/mol) although showing almost the same overall value ($\Delta$G$^{\ddagger }$ $\approx$ 120 kJ/mol at both pH values). The explanation of this apparent paradox relies on the different temperature-dependence of the corresponding $k_{obs}$ (higher at pH 3 than at pH 4), thus indicating a temperature-inhibitory effect at higher acidities. It is not easy to find a clear explanation of this pH-dependence because it is unknown how pH affects the overall $k_{obs}$. A plausible reason could be the pH-dependent % molar distribution of the sulfur-based nucleophilic species ($\hbox {HSO}_3^-$ $_{(aq)} / \hbox {SO}_2$ $_{(aq)}$ and/or radical anion sulfur-centred species as $\hbox {SO}_3^{\cdot -}$). Another striking outcome is the great contribution of the negative activation entropy term T$\Delta \hbox {S}^{\ddagger }$ ($-72.3$ kJ/mol at $30\, ^\circ \hbox {C}$, pH 3) to the overall rate of conversion (Table 1). Although negative values for $\Delta \hbox {S}^{\ddagger }$ are largely expected in an associative mechanism in which two reaction partners form a single activated complex, the large negative values here observed indicate that in the rate-determining step there is a very large loss of molecular degrees of freedom of reactant species going toward the higher transition state of the process. From a practical point of view, the predominance of this entropic term leads to the consequence that by increasing the reaction temperature, the conversion percentage of **1** $\rightarrow$ **5** does not increase as much as expected from its total free energy of activation $\Delta$G$^{\ddagger }$.

To our knowledge, this was the first time that monomeric sulfonated flavanols were proved to be produced by hydrogen sulfite attack on monomeric flavanols. Indeed, to date, epicatechin 4$\beta$-sulfonate (**5**) was detected only by starting from oligomeric mixtures present in wine^25^, apple extract^26^, grape skin extract^27^ or bark extract^28^. Therefore, our result clearly indicates that tannin de-polymerisation is not mandatory for the production of sulfonated flavanols. As further support, the results of our control experiments, carrying out the same kinetic runs without $\hbox {SO}_2$ addition, do not indicate any formation of dimeric forms.

**Figure 5:**
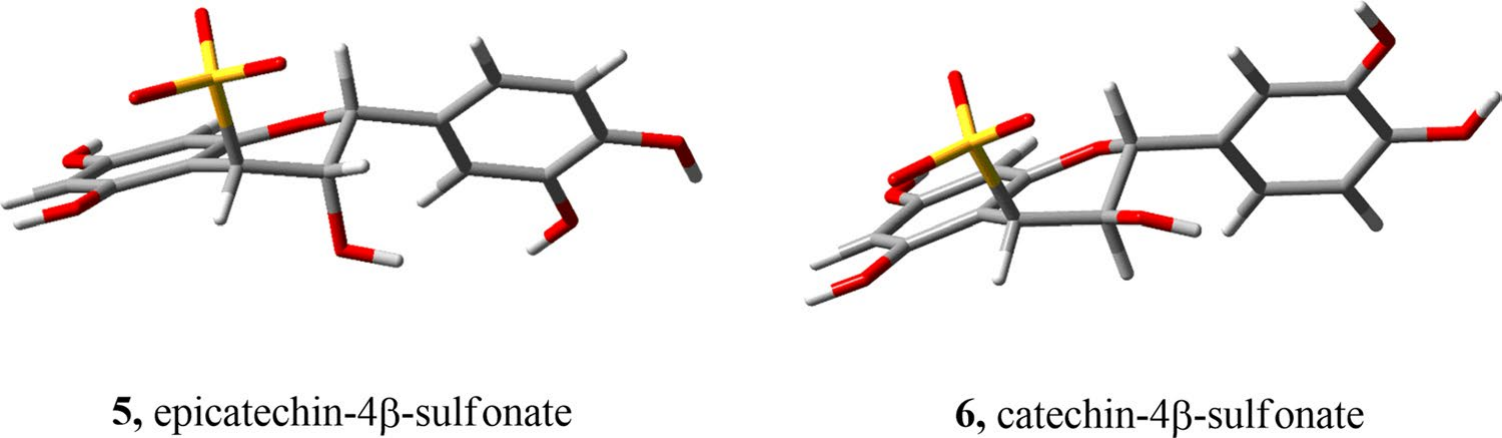
3D geometry optimised structures of **5** and **6** as obtained by molecular mechanics (GMMX) calculations (energy minimisation).

The kinetic parameters of the sulfonation process **2** $\rightarrow$ **6** were also obtained following the same approach (Fig. 4c, d and Table 1). The relative stereochemistry of catechin 4$\beta$-sulfonate (**6**) was established by NMR analysis at the end-point of the reaction **4** $\rightarrow$ **6**. In the $^1$H-NMR spectrum of this mixture the signals of **6** appear clearly separated from those of the expected hydrolytic product **2** and from lower signals attributable to **8**. The *cis*-(3,4) stereochemistry and thus the $\beta$ position of the sulfo group at C(4) was established by the $^3$J coupling analysis where the inter-proton coupling constant, $^3$J(3,4) was found to be 4.9 Hz. Since H–C(3) occupies an axial position in catechin-based metabolites, this value is only in accordance with H–C(4) in an equatorial position, i.e $^3$J(3ax,4eq); indeed, the other relationship would be reflected by a $^3$J(3ax,4ax) expected to be much higher ($\approx$ 10 Hz). Thus, the sulfo group lies in a $\beta$-pseudo axial position of the ring C, i.e. we are dealing with (2R, 3R, 4S)-4$\beta$-sulfocatechin (**6**) (Fig. 5). This result is in fair agreement with molecular mechanics calculations (MM-GMMX) which indicated for the minimised structure of **6** a dihedral angle HC(3)–C(3)–C(4)–HC(4) $\sim 46^\circ$, perfectly compatible with the observed $^3$J(3ax,4eq) value (4.9 Hz). Worth of note, according to MM calculations, **6** is thermodynamically more stable than its diasteroisomer with the $\alpha$-sulfo group at C(4).

As observed above in the **1** $\rightarrow$ **5** conversion, also in the investigation of the process **2** $\rightarrow$ **6** the extracted ESI(−) mass chromatogram of the anion at m/z 369.03 (C$_{15}\hbox {H}_{13}$O$_9$S$^-$) showed the presence of three other minor isobaric species, besides the major **6**, at different retention times. MS/MS measurements suggested the presence of minor amount of the diasteroisomer of **6** (4$\alpha$-sulfo analogue, $\sim 15\%$ of the major **6** at pH 3) and of minor sulfonated products at ring B ($\sim 20\%$ of the major **6** at pH 3); similarly to what observed with epicatechin, also in catechin the presence of ring-B sulfonated products becomes comparable with the major **6** at pH 4. We describe here only the kinetics of formation of **6** because the formation of the others was so low at lower temperature to be difficult to detect and in any case difficult to quantitate also at higher temperature.

The conversion percentage of **6** increases of a magnitude order from $23\, ^\circ \hbox {C}$ (0.02%/day) to $60\, ^\circ \hbox {C}$ (0.24%/day), leading to a rough estimate of less than 10% conversion/year of **2** $\rightarrow$ **6** at the usual non-optimal wine storage temperatures (25–$30\, ^\circ \hbox {C}$), suggesting a slightly higher sulfonation reactivity of epicatechin with respect to catechin. Even here, as observed for **1** $\rightarrow$ **5**, the value is possibly underestimated since **6** is not the only observed monosulfonated product. Moreover, as observed for **1**, no significant differences were observed in the pH-dependence of the rate constants; concerning their temperature-dependence, this process shows only a slightly higher activation energy at pH 3 than at pH 4 ($\Delta \Delta \hbox {H}^\ddagger \sim 4$ kJ/mol) but not so significant as observed for **1** $\rightarrow$ **5** reaction ($\Delta \Delta \hbox {H}^\ddagger \sim 9$ kJ/mol). Great resemblance to **1** $\rightarrow$ **5** reaction was found, however, in the high contribution of the negative activation entropy term (T$\Delta$S$^{\ddagger } = -71.4$ kJ/mol at $30\, ^\circ \hbox {C}$, pH 3) to the overall free energy of activation ($\Delta \hbox {G}^{\ddagger } \sim 123$ kJ/mol at $30\, ^\circ \hbox {C}$, pH 3).

**Figure 6:**
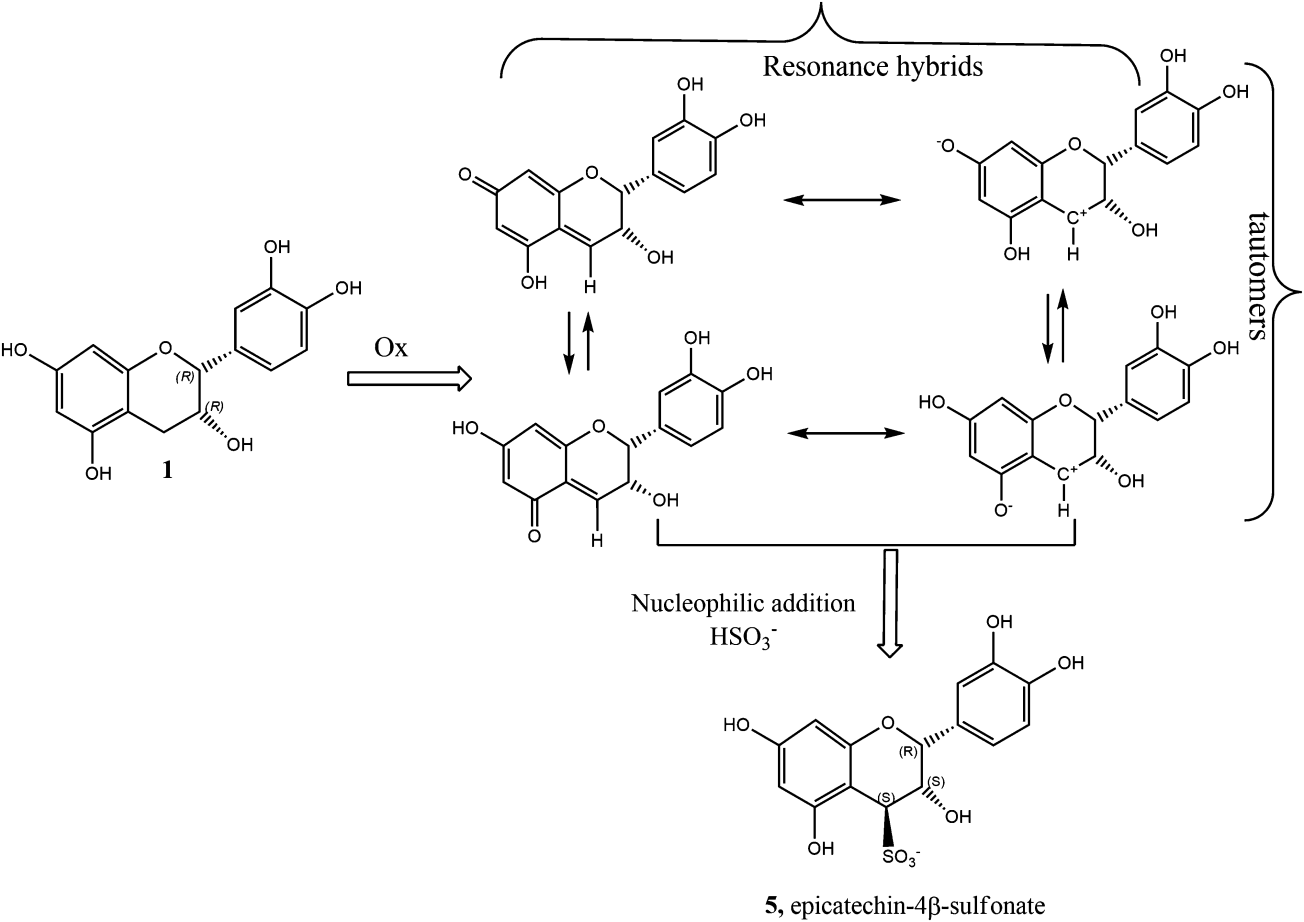
Proposed mechanism for the sulfonation of epicatechin **1**.

Our proposal of a plausible route to 4-sulfonated derivatives from monomeric flavanols is outlined in Fig. 6 for the reaction **1** $\rightarrow$ **5**. In our view, a preliminary (possibly rate-determining) oxidation step is required to generate the quinone-methides **15**. The resonance zwitterionic hybrids (on the right in Fig. 6) of the spin-paired *p*-quinone or *o*-quinone methides (on the left in Fig. 6) could also play a role. Of course, the issue here is that the chemical species that could play the role of oxidant required in the first step are not known. We advance here the hypothesis that the sulfite radical anion $\hbox {SO}_3^{\cdot -}$, produced by the one-electron oxidation of sulfite or bisulfite ions in presence of O$_2$ or any other oxidising species present in solution could be the oxidising agent^40^. The $\hbox {SO}_3^{\cdot -}$ radical is a sulfur-centred radical which can act as an oxidant or reductant and, like most other radicals, may engage in hydrogen abstraction leading to quinone methides **15** or addition to double bonds leading to **5**. Preliminary data in our hands clearly demonstrated that in the presence of butylated hydroxytoluene (BHT), a well known radical scavenger, the conversion percentage of **5** becomes significantly lower than that observed in untreated reaction systems (for epicatechin, the conversion decreased by 40% at pH 3 and $60\, ^\circ \hbox {C}$), thus a radical route cannot be ruled out.

As underlined in the introduction section, kinetic investigations dealing with the sulfonation of monomeric flavanols cannot neglect the competitive processes affording the isomerisation products^41^, whereby **1** is converted in *ent*-**2** and **2** is converted in *ent*-**1** (Fig. 3). Since MS data are not sensitive to the absolute configuration of **1** (= *ent*-**1** ) or **2** (= *ent*-**2**), our chromatographic run allows to follow, besides the formation of their sulfo-derivatives **5** (or **6**) also that of their isomers *ent*-**2** (or *ent*-**1**) as **2** (or **1**) themselves. Luckily, since **1** and **2** have different retention times in our chromatographic conditions, we had the opportunity to evaluate whether and to what extent this isomerisation process occurs. Actually, the observed rate constants of isomer formation were found to be quite small even at the highest investigated temperatures but clearly not negligible with respect to the formation of the sulfo-derivatives **5** and **6** (Supplementary Table S1 online). Indeed, for the process **1** $\rightarrow$ *ent*-**2** (= **2**) (pH 3, $60\, ^\circ \hbox {C}$), the specific rate of isomerisation was found to be almost identical to the specific rate of sulfonation, and at higher pH (pH 4, $60\, ^\circ \hbox {C}$) the former was found to be even significantly higher (1.2 fold change) than the latter. The overall kinetic barrier of isomerisation was found to be slightly higher ($\Delta \Delta$G$^\ddagger \ge 4$ kJ/mol) for **2** $\rightarrow$ *ent*-**1** conversion than for **1** $\rightarrow$ *ent*-**2** conversion, but dealing with a single step first order process, this could simply reflect the higher thermodynamic stability of catechin over epicatechin due to the more stable *trans*-diequatorial position of the substituents at C(2) and C(3) on the former. The overall quality of our kinetic data (mainly in term of reproducibility) does not allow to dissect the $\Delta$G$^\ddagger$ values of these isomerisations into enthalpic and entropic relative contributions, but it is largely expected that, since **11** and **13** are key intermediates in the isomerisation mechanism of **1** and **2** (Fig. 3), this process should be $\Delta \hbox {H}^\ddagger$-controlled (bond breaking required). As a further support for our guess, it is well known that isomerisations are fast processes only at temperatures $\ge$ 80 $^\circ \hbox {C}$^36,39^.

### Kinetics of sulfonation processes of dimeric flavanols **3** and **4**

**Figure 7:**
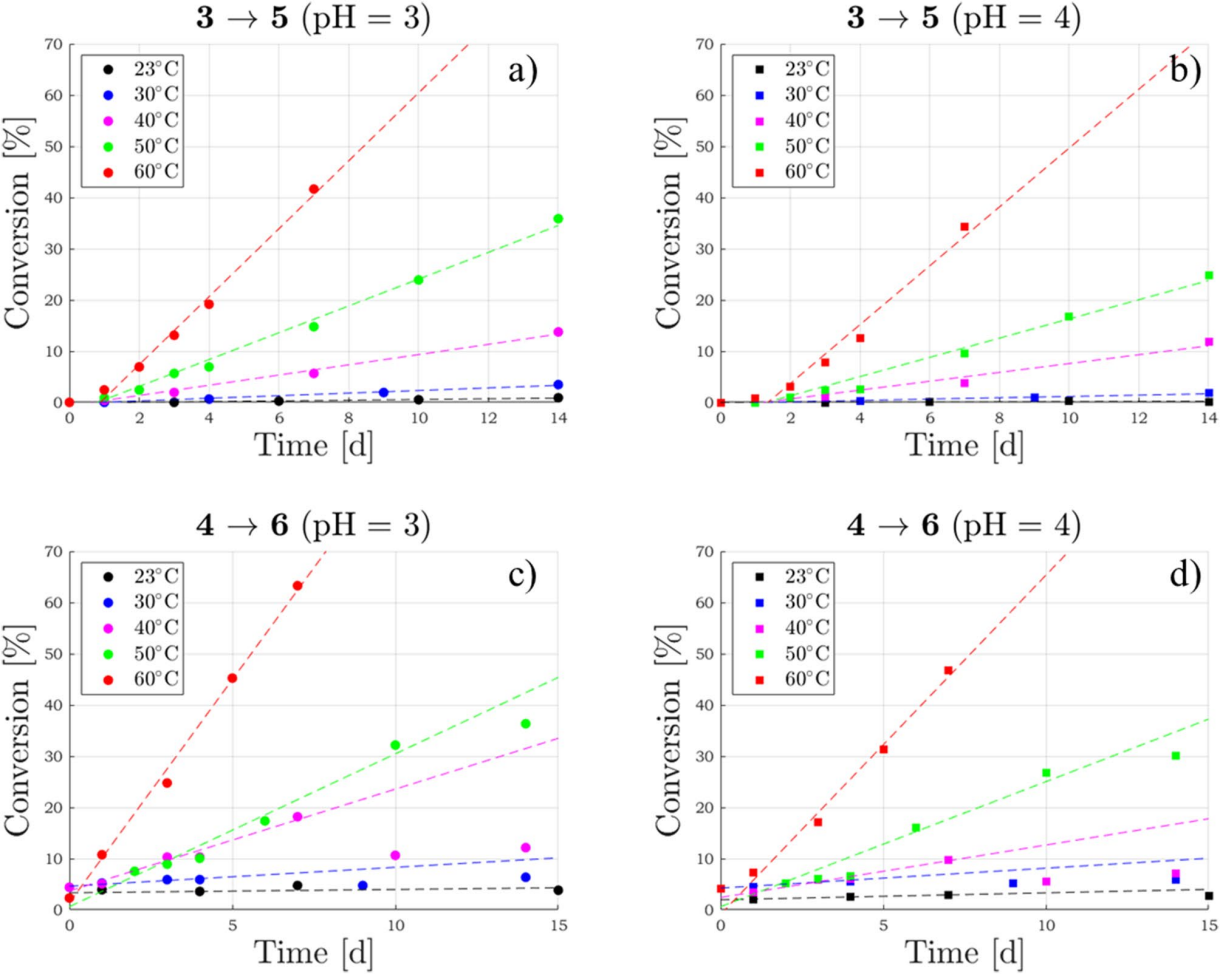
Comparison of the different temperature kinetics of epicatechin 4$\beta$-sulfonate (**5**) and catechin 4$\beta$-sulfonate (**6**) formation starting, respectively, from procyanidin B2 (**3**) **(a, b)** and procyanidin B3 (**4**) **(c, d)** at pH 3 and 4. R$^2$ coefficients can be found in Supplementary Table S3 online.

In order to compare the rate and overall yield of the sulfonation processes of dimeric flavanols **3** and **4**, we followed the same approach as outlined above for the monomeric **1** and **2**, carrying out the LC-MS measurements at different times, pH and temperature values of the sulfonation processes of **3** $\rightarrow$ **5** and **4** $\rightarrow$ **6** (Fig. 7). Worth of note, only traces or very low concentration of the sulfonated dimers **7** and **8** were detected in our LC-MS measurements (Supplementary Fig. S3, S4 online), pointing out that acidic interflavanic bond cleavage of **3** and **4** is much faster than their sulfonation (Fig. 2). This outcome could also explain why epicatechin 4$\beta$-sulfonate is the major sulfonated flavanol found in wine (Supplementary Fig. S5 online), since epicatechin is the predominant extension unit in tannins^42^. The comparison indicates quite similar specific rates for the process **3** $\rightarrow$ **5** and **4** $\rightarrow$ **6** but they are very different from those observed in the corresponding monomeric flavanols (**1** $\rightarrow$ **5** and **2** $\rightarrow$ **6**). In the proposed plausible mechanism^27^, the breakage of the C(4)-C(8) link in **3** (or **4**) is the rate-determining step leading to monomeric **1** (or **2**) through the C(4) carbocations **9** (or **10**), as oulined in Fig. 2. Since for dimeric flavanols the % conversion into **5** (or **6**), during the reaction time, was not negligible with respect to starting reactants **3** (or **4**), we assumed a pseudo first order kinetics model based on the simple equation $[\mathbf{5} ]_t = [\mathbf{3} ]_0(1-exp\{(-k_{obs} * t\})$. There are two hypothesis/approximations in this approach. The first one deals with the mass balance of the reacting substrate (**3** or **4**) that we assume could afford only unique sulfonated products (**5** or **6**). The second one is based on the assumption that monomeric flavanols themselves (**1** or **2**) cannot be subjected to further sulfonation in the reaction system. We are confident that the second condition is largely fulfilled since the sulfonation of monomeric flavanols is much slower than that of dimeric flavanols, i.e. the detected sulfonated products **5** or **6** can be considered as deriving only from reacting dimers and not from **1** or **2** in our reacting system. The first one is a more subtle hypothesis, but we have experimental evidence (see Supplementary Fig. S1 online) that other isobaric sulfonated products, detected in the sulfonation of monomeric flavanols, are not present in the reacting mixture.

By knowing the initial concentration of procyanidins [**3**]$_0$ (or [**4**]$_0$) and measuring [**5**]$_t$ (or [**6**]$_t$) at different times for given pH/T values, we evaluated the $k_{obs}$ values (Table 2) from the best-fitting of the curves ln{($[\mathbf{3} ]_0 - [\mathbf{5} ]_t) /[\mathbf{3} ]_0$} versus time for the process **3** $\rightarrow$ **5** (or ln{($[\mathbf{4} ]_0 - [\mathbf{6} ]_t) /[\mathbf{4} ]_0$}) versus time for the process **4** $\rightarrow$ **6**). On the other hand, reliable estimates of kinetic barriers (Table 2) were also obtained from the temperature dependence of these values, following Arrhenius and Eyring kinetic models. Significant differences with respect to monomeric flavanols were found both on the pH- and temperature-dependence of the corresponding $k_{obs}$: as above reported, the specific rate of sulfonation process **3** $\rightarrow$ **5** is much higher (about 20 times) than that of process **1** $\rightarrow$ **5**. Moreover, $k_{obs}$ are higher in dimeric flavanols at pH 3 (1.5 times) than at pH 4; a result largely expected, since the interflavanic bond cleavage of the C(4)–C(8) link between the two epicatechin units in **3** (or catechin units in **4**) is acid-catalysed^42^. In fact, the slightly higher values for the observed rate constants (Table 2) at lower pH for the conversion **3** $\rightarrow$ **5** are in agreement with previous kinetics data reported by Hemingway et al.^30^; curiously in this paper the authors have found higher activation energies for faster processes. Anyhow, the chemical conditions used by these authors to carry out the cleavage of the interflavanic bond is quite different from our experimental setup and the kinetic equations could depend from [H$_{3}$O$^{+}$] in a different way. This outcome is thus reflected in the higher activation energy E$_A$ (or $\Delta \hbox {H}^\ddagger$) at pH 4 with respect to pH 3, being $\Delta$E$_A$ = E$_A$(pH 4) - E$_A$(pH 3) $\approx$ $\Delta \Delta \hbox {H}^\ddagger$ $\approx$ 5.0 kJ/mol. More importantly, the % conversion of procyanidin B2 (**3**) into epicatechin 4$\beta$-sulfonate (**5**) after 7 days ($60\, ^\circ \hbox {C}$, pH 3) was about 40%, thus much higher than the observed conversion in the sulfonation of monomeric **1**. Interestingly, our Arrhenius (or Eyring) analysis for the process **3** $\rightarrow$ **5** clearly shows that this rate-enhancement is not due to lower E$_A$ (or $\Delta \hbox {H}^\ddagger$), as expected by an activation enthalpy–controlled process. In fact, the evaluated $\Delta$E$_A$ = E$_A$(**3** $\rightarrow$ **5**) – E$_A$(**1** $\rightarrow$ **5**) $\approx$ $\Delta \Delta \hbox {H}^\ddagger$ $\ge +40$ kJ/mol suggests an opposite trend of the observed rates. The apparent paradox is easily resolved by taking into account the negative entropic activation contribution (T$\Delta$S$^\ddagger$) that is much higher for the sulfonation of **1**. In other words, whilst the process **1** $\rightarrow$ **5** is strongly $\Delta$S$^\ddagger$-controlled, the process **3** $\rightarrow$ **5** turns out to be essentially as a $\Delta \hbox {H}^\ddagger$-controlled process, which hints at a completely different reaction mechanisms of the sulfonation reactions with regard to monomeric as opposed to dimeric flavanols.

**Table 2:** Observed rate constants and kinetic parameters of the processes **3** $$\rightarrow$$ **5** and **4** $$\rightarrow$$ **6** at different pH; $$^{a)}$$ values calculated at $$30\, ^\circ \hbox {C}$$.

Process	T ($^\circ \hbox {C}$)	$k_{obs}$ (day$^{-1}$)	E$_A$ (kJ/mol)	$\Delta {H}^{\ddagger}$ (kJ/mol)	${}^{\mathrm{a}} \Delta {S}^{\ddagger}$ [kJ/(mol K)]	${}^{\mathrm{a}} \Delta {G}^{\ddagger}$ (kJ/mol)
**3** $\rightarrow$ **5** (pH = 3)	23	$(1.6 \pm 0.3) \times 10^{-3}$	91.0 ± 0.3	88.4 ± 0.4	$-0.094 \pm 0.001$	117.1 ± 0.5
	30	$(3.7 \pm 0.8) \times 10^{-3}$				
	40	$(11.8 \pm 5.2) \times 10^{-3}$				
	50	$(34.5 \pm 2.6) \times 10^{-3}$				
	60	$(95.1 \pm 15.4) \times 10^{-3}$				
**3** $\rightarrow$ **5**(pH = 4)	23	$(0.7 \pm 0.1) \times 10^{-3}$	101.9 ± 9.9	99.3 ± 9.9	$-0.063 \pm 0.032$	118.4 ± 13.9
	30	$(2.4 \pm 0.8) \times 10^{-3}$				
	40	$(10.9 \pm 2.7) \times 10^{-3}$				
	50	$(23.6 \pm 1.8) \times 10^{-3}$				
	60	$(79.8 \pm 17.5) \times 10^{-3}$				
**4** $\rightarrow$ **6** (pH = 3)	23	$(1.9 \pm 0.5) \times 10^{-3}$	87.0 ± 13.3	84.4 ± 13.3	$-0.105 \pm 0.043$	116.2 ± 18.6
	30	$(6.6 \pm 0.5) \times 10^{-3}$				
	40	$(20.6 \pm 2.1) \times 10^{-3}$				
	50	$(32.1 \pm 5.3) \times 10^{-3}$				
	60	$(126.4 \pm 2.3) \times 10^{-3}$				
**4** $\rightarrow$ **6** (pH = 4)	23	$(1.4 \pm 0.4) \times 10^{-3}$	89.4 ± 4.7	86.8 ± 4.7	$-0.100 \pm 0.015$	117.1 ± 6.6
	30	$(4.0 \pm 1.9) \times 10^{-3}$				
	40	$(11.0 \pm 2.3) \times 10^{-3}$				
	50	$(28.4 \pm 5.6) \times 10^{-3}$				
	60	$(92.5 \pm 24.4) \times 10^{-3}$				

Moving along similar lines, we were able to obtain the observed rate constants and the kinetic parameters (Table 2) for the process **4** $\rightarrow$ **6**. As an example, at pH 3/$60\, ^\circ \hbox {C}$, the specific rate constants of this process were found to be slightly higher (1.4 times) than at pH 4/$60\, ^\circ \hbox {C}$, about 50 times faster than those observed for **2** $\rightarrow$ **6** at the same pH/T values, but similar to those observed for the process **3** $\rightarrow$ **5** (1.3 time faster), still at the same pH/T values. The above mentioned considerations about the relative contribution of $\Delta \hbox {H}^\ddagger$ or $\Delta$S$^\ddagger$ to the overall kinetic barrier $\Delta$G$^\ddagger$ for the process **3** $\rightarrow$ **5** hold true also for the process **4** $\rightarrow$ **6**, the latter essentially being a process under $\Delta \hbox {H}^\ddagger$-control. Thus, to summarise our results, the sulfonation of flavanol dimers occurs much faster than that of monomeric flavanols, no matter the relative stereochemistry of their chiral centres, giving further support to the mechanism (Fig. 2) where the rate-determining step should be represented by the formation of C(4) carbocation intermediates **9** (or **10**) after acid-catalysed interflavanic bond cleavage, followed by a fast capture of nucleophilic $\hbox {HSO}_3^-$ ion species leading to **5** (or **6**). In order to shed light on the mechanistic details of the complex chemistry of dimeric flavanols, we evaluated the kinetic parameters of the interflavanic bond cleavage itself by following the rate of appearance of **1** in the process **3** $\rightarrow$ **1** + **5** and the rate of appearance of **2** in the process **4** $\rightarrow$ **2** + **6** (Supplementary Table S2 online). Concerning the process **3** $\rightarrow$ **1** + **5**, a simple comparison of the independently evaluated observed rate constants for the time-appearance of **1** and **5** at any fixed temperature and pH (e.g. $60\, ^\circ \hbox {C}$, pH 3) indicated that they are produced at almost the same specific rate. However, at lower temperatures (e.g. 23 $^\circ \hbox {C}$, pH 3) the specific rate of formation of **1** is almost a magnitude order higher than the formation of **5**. Even more surprisingly was that an even simpler comparison between the change in conversion percentage of **1** and **5** at different temperatures at any fixed time and pH (e.g. 7 days, pH 3) leads to conclude that whilst the process **3** $\rightarrow$ **5** is mainly under activation enthalpy-control ($\Delta \hbox {H}^\ddagger$ $\approx$ 89 kJ/mol, T$\Delta$S$^\ddagger$ $\approx$ − 32 kJ/mol), for the process **3** $\rightarrow$ **1** the contribution of the activation entropy is much more pronounced ($\Delta \hbox {H}^\ddagger$ $\approx$ 49 kJ/mol, T$\Delta$S$^\ddagger$ $\approx$ − 72 kJ/mol). The same difference is even more pronounced at pH 4 ($\Delta \hbox {H}^\ddagger$ $\approx$ 89 kJ/mol, T$\Delta$S$^\ddagger$ $\approx$ − 22 kJ/mol for **3** $\rightarrow$ **5**; $\Delta \hbox {H}^\ddagger$ $\approx$ 54 kJ/mol, T$\Delta$S$^\ddagger$ $\approx$ − 69 kJ/mol for **3** $\rightarrow$ **1**) and also maintained in the kinetics of procyanidin B3 (**4** $\rightarrow$ **2** + **6**), thus indicating a general feature of this pathways. In other words, although procyanidin interflavanic bond cleavage occurs on almost the same time-scale as sulfonation (only with a higher relative rate at lower temperatures) indicating a very similar overall kinetic barrier ($\Delta$G$^\ddagger$ $\approx$ 121 ± 1 kJ/mol) as expected for the mechanism outlined in Fig. 2, the significantly different contributions of the enthalpy and entropy factors to this kinetic barrier clearly indicate that **1** and **5** are produced from **3** following different pathways; this outcome casts doubts on the proposed mechanism^27^ relying on the carbocation **9** as key intermediate for the formation of both **1** and **5** due to different temperature-dependence of the appearance of **1** and **5** in the same reaction system. Further investigations are necessary to shed light on the mechanistic details of these reactions but, with the kinetic data in our hand, it seems reasonable to suggest that the rate-determining step of sulfonation in procyanidins could be a concerted bimolecular process instead; this guess could also explain the high stereoselectivity of the $-\hbox {SO}_3^-$ group attack at C(4) difficult to rationalise by assuming C(4) as a planar carbocationic centre. Whereas the mechanism of procyanidin acid-catalysed interflavanic bond cleavage has been deeply investigated^29–31,43^, studies on the sulfonation mechanism are completely lacking. We planned to fill this void with this investigation, but further efforts are needed to clarify several other aspects. In particular, a new mechanistic proposal should be able to explain the origin of the stereoselectivity of the ring-C sulfonation of both monomeric and dimeric flavanols (the sulfo-group attacks predominantly on the $\beta$ side, no matter the chirality at C(3) of the considered flavanol). Since the presence of radical (anionic) S(V) species cannot be ruled out in our reacting system of monomeric flavanols, care must be taken to identify the main radical producing agent, the propagation and termination of this chain reaction^40^. It is important to point out that our results on monomeric and dimeric flavanols reactivity towards sulfur dioxide shed some light on wine tannins chemistry; although this paper describes kinetics of reactions in a model solution, in terms of relative concentrations the reaction system was selected to be similar to actual wine systems. With this in mind, we found that flavanol sulfonation occurred much faster on dimeric substrates, which could be a major route also in tannin sulfonation systems. This outcome could further explain why epicatechin 4$\beta$-sulfonate is the major sulfonated flavanol found in wine, since epicatechin is the predominant extension unit, followed by epigallocatechin^42^. Therefore, catechin 4$\beta$-sulfonate detection and quantification in wine should be more challenging, since the direct sulfonation reaction and the concentration of proanthocyanidins with catechin extension unit are low. However, we cannot exclude their presence in wine or other tannin-rich food to which $\hbox {SO}_2$ is added. Next to the previous knowledge that **5** production is favoured by storage at higher temperature we found that, at the usual non-optimal wine storage temperatures (25-$30\, ^\circ \hbox {C}$), monomer sulfonation occurs 5 to 10 times faster on dimeric substrates than on monomeric ones. These data are in agreement with the results obtained by wine storage and wine ageing experiments^22,25^. In view of these results, we can assume that if a wine initially contains only dimeric procyanidins, every $^\circ \hbox {C}$ degree increment during storage (in a range of temperatures between 23 and $30\, ^\circ \hbox {C}$) corresponds to an increment of $15 \div 20 \%$ in the formation of monomeric sulfonated flavanols. This result demonstrates the paramount importance of temperature in the production of sulfonated flavanols and that the further modelling of this reaction could provide a key to calculate wine chemical age.

## Conclusions

Our kinetic investigation of the sulfonation of monomeric and dimeric flavanols in a wine-model reacting system, has allowed us to obtain a reliable estimate of pH- and temperature-dependence of the rate constants, not only of this process, but also of several competitive processes occurring in wines. In particular, we found that direct sulfonation starting from catechin and epicatechin is possible, even if the process is slower when compared to the corresponding one starting from procyanidins; the latter, no matter the type of monomeric units they are built from, are subject to extensive interflavanic bond cleavage, sulfonation and monomer isomerisation processes whose relative contribution to their overall disappearance was carefully evaluated by quantitative LC/MS measurements for the first time in this study. With these data in hand, it seems that sulfonation occurs as competitive process, along the generally accepted path of procyanidins acidic-catalysed interflavanic bond cleavage relying on fast/reversible protonation of hydroxyl on ring A in the terminal unit followed by a rate-determining step leading to a planar carbocation at C(4) of the upper flavanol unit. However, the high $\beta$ stereoselectivity of the attack of $\hbox {SO}_2$ to C(4) seems difficult to explain assuming a planar carbocation as key intermediate. Actually, the existence of this intermediate along the reaction path has never been proved, not even in pure hydrolytic processes; surely, any role of this intermediate in the sulfonation of monomeric flavanols must be ruled out. Moreover, our data show a completely different dependence of the kinetic barrier of the sulfonation processes of monomeric and dimeric flavanols, where the former results under activation entropy-control whilst the latter under activation enthalpy-control, a clear indication of different reaction mechanisms. A plausible mechanistic proposal for the sulfonation of monomeric flavanols is here also proposed. This new knowledge provides essential information in order to better understand tannin chemistry in food and predict or model the chemical/sensorial behaviour of wine or other food rich in proanthocyanidins.

## Methods

All the reactions took place in 2 mL HPLC amber vials so it was possible to analyse them directly through UPLC-DAD-QTOF-MS. The initial concentration for all the reaction were (0.35 ± 0.01) mM, for epicatechin and catechin, (0.039 ± 0.001) mM for procyanidin B2 and (0.043 ± 0.002) mM for procyanidin B3. The concentrations were chosen to be close to the typical wine concentration. The model wine solution was prepared with purified Milli-Q water, 5$\%$ ethanol, and 1642 mg/L sodium metabisulfite, while the pH was adjusted with formic acid. Sodium metabisulfite was added in molar excess with respect to the flavanols ($\sim$1:50 for monomers, $\sim$1:400 for dimers). For each reactant the reactions were carried out at two pH values (pH 3 and 4) and at five temperatures (23, 30, 40, 50 and $60\, ^\circ \hbox {C}$). The same set of reactions were repeated as control without the addition of sodium metabisulfite, thus 80 runs were investigated. Finally, for the sodium metabisulfite reaction four vials were prepared, filled up at maximum, for each epicatechin and catechin reaction, and three vials for each procyanidin B2 and procyanidin B3 reaction. One vial from each reaction was analysed at the end of the experiment, while all the others were analysed at time zero and at least four more times during the 14 days of each experiment. Two vials were prepared for each control reaction and they were analysed at least four times during the 14 days of each experiment. The reactions were monitored by a Waters Acquity UPLC coupled via an electrospray ionisation (ESI) interface to a PDA Acquity detector and a Synapt HDMS QTOF MS (Waters, Manchester, UK) operating in W-mode and controlled by MassLynx 4.1, according to previously described parameters^25,44^. Before going to the mass spectrometer, the injected samples were passing through the Photodiode Array (PDA) detector cell, which was able to acquire their UV spectra from 210 to 490 nm (resolution 1.2 nm and sample rate 10 points/s).

Extra samples with BHT addition were prepared, using BHT in the same molar excess conditions as sodium metabisulfite, and analysed with the same described method; data were compared between same temperature, pH and elapsed time. Calibration curves were constructed for each standard at eight to ten concentration levels (approximately from 0.2 to 200 mg/L) by using the mobile phases (95$\%$ A and 5$\%$ B) for the dilutions. The calibration curves and the quantification were made by using the TargetLynx tool of MassLynx. *ent*-catechin and *ent*-epicatechin were relatively quantified with the calibration curve of their corresponding enantiomer; epicatechin 4$\beta$-sulfonated and procyanidin B2 4$\beta$-sulfonated were prepared according to^26^; catechin 4$\beta$-sulfonated was quantified as epicatechin 4$\beta$-sulfonated.

MS/MS measurements were carried out on the same UPLC-QTOF-ESI(−)MS/MS system, using the initial quadrupole to isolate the target ions at m/z 369.03 and the TOF-MS as ion analyser; instrumental parameters were adjusted in order to achieve the best fragmentation pattern.

Data analysis was made by using Arrhenius and Eyring models, the graphs were made with Matlab and Microsoft Excel.

$^1$H-NMR spectrum of crude end-point of the **4** $\rightarrow$ **2** + **6** process dissolved in D$_2$O were recorded at 300 K on a Bruker-Avance 400 MHz NMR spectrometer, with a 5-mm BBI probe outfit with pulsed-gradient field utility. The $^1$H-90$^\circ$ proton pulse length was 9.3 $\mu$s with a transmission power of 0 dB. Probe temperature was maintained at 300 ± 0.1 K by a Bruker B-VT 1000 variable temperature unit. Calibration of the chemical shift scale ($\delta$) was carried out on the residual proton signal of the D$_2$O at $\delta$H 4.67 ppm. The following measurements were performed (with the acquired information): $^1$H-NMR (proton chemical shifts and scalar couplings J); $^1$H-$^{13}$C HSQC (proton-carbon one-bond correlation); $^1$H-$^{13}$C HMBC (proton-carbon multiple-bond correlation). NMR spectra were analysed by MestreNova 12.0 software (Mestrelab research S.L.2012, Escondido, CA).

Molecular mechanics calculations on compounds **1**, **2**, **5** and **6** were carried out by GMMX (implemented in Gaussian 16 suite of programs) both in optimisation and in conformer search mode; from the latter we were able to determine the most stable conformations of the studied molecules and their relative population distribution. Alternative epimers at C(4) of **5** and **6** were also investigated.

## Supplementary information

Supplementary Information. (PDF 856 kb)

## Data Availability

The datasets generated during and/or analysed during the current study are available from the corresponding author on reasonable request.
