# Supplementary material for: Kinetic investigations of sulfite addition to flavanols
Source: Sci Rep. 2020 Jul 30;10:12792. doi: 10.1038/s41598-020-69483-0 (PMC7393157; doi:10.1038/s41598-020-69483-0)
Supplement: Supplementary file 1 — Supplementary Information. (PDF 856 kb) [file 41598_2020_69483_MOESM1_ESM.pdf]

Kinetic investigations of sulfite addition to flavanols

Federico Bonaldo[1,2], **Graziano Guella**[2,*], **Fulvio Mattivi**[1,2], **Daniele Catorci**[2], **and Panagiotis Arapitsas**[1,*]

[1]Department of Food Quality and Nutrition, Research and Innovation Centre, Fondazione Edmund Mach (FEM), San Michele all'Adige, Italy

[2]Bioorganic Chemistry Laboratory, Department of Physics, University of Trento, Trento, Italy

[*]corresponding authors: panagiotis.arapitsas@gmail.com, graziano.guella@unitn.it

Supplementary Material

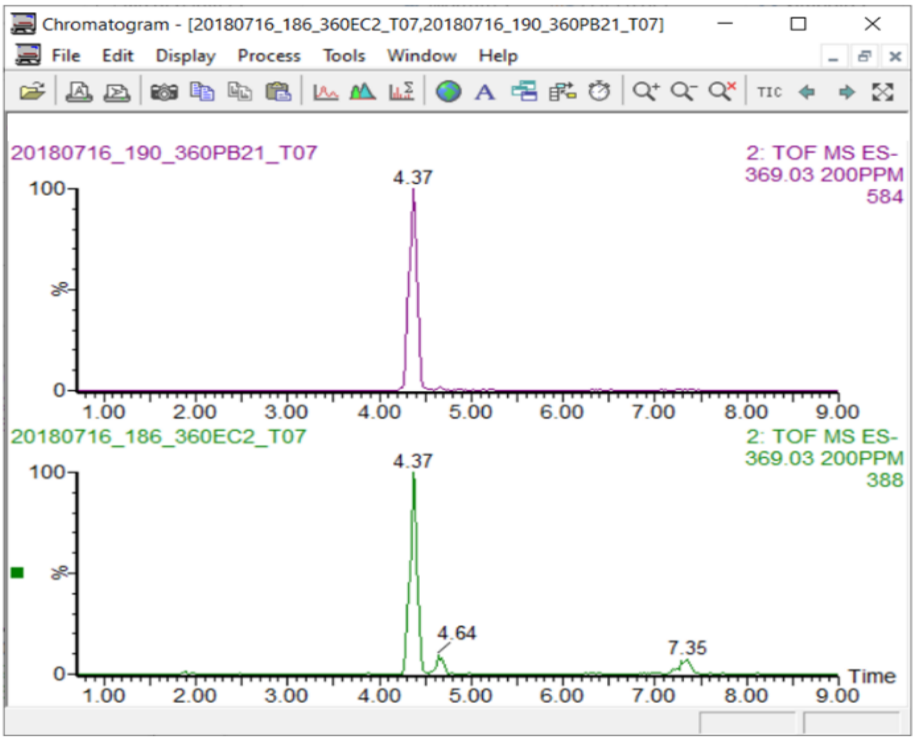

Supplementary Figure S1. Top: SIM of *m/z* 369.03 extracted from the chromatogram of procyanidin B2 kinetics (pH 3, T = 60 °C, after 7 days); **Bottom**: SIM of *m/z* 369.03 extracted from the chromatogram of epicatechin kinetics (pH 3, T = 60 °C, after 7 days). Peak at RT = 4.37 min represents epicatechin 4β-sulfonate (**5**), while the minor peaks present only in monomeric starting reaction correspond to isomers of **5**; this is a clear evidence of the sulfonation occurring only on C(4) starting from dimers.

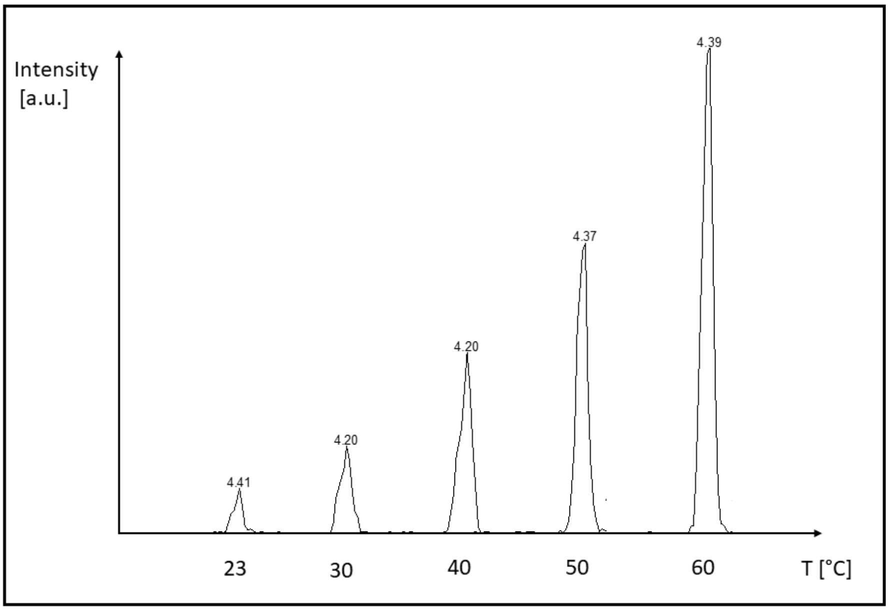

Supplementary Figure S2. LC-MS SIM peak evolution as function of temperature at a fixed time; chromatographic peaks correspond to epicatechin 4β-sulfonated compound (**5**, m/z = 369.03 and RT ~ 4.3 min) at pH = 3, after 7 days. Peak area integration allowed us to obtain concentration of the investigated sulfonated product.

PROCESS	T [°C]	k_{obs} [d^{-1}]		PROCESS	T [°C]	k_{obs} [d^{-1}]
**1** → *ent*-**2** pH = 3	23	$(2.5 \pm 0.7)x10^{-4}$		**2** → *ent*-**1** pH = 3	23	$(7.2 \pm 0.1)x10^{-5}$
	30	$(5.3 \pm 0.1)x10^{-4}$			30	$(11.3 \pm 3.1)x10^{-5}$
	40	$(15.4 \pm 2.1)x10^{-4}$			40	$(32.3 \pm 5.8)x10^{-5}$
	50	$(35.4 \pm 5.2)x10^{-4}$			50	$(84.3 \pm 3.6)x10^{-5}$
	60	$(57.7 \pm 3.6)x10^{-4}$			60	$(273.0 \pm 20.5)x10^{-5}$
**1** → *ent*-**2** pH = 4	23	$(4.9 \pm 0.9)x10^{-4}$		**2** → *ent*-**1** pH = 4	23	$(7.9 \pm 0.6)x10^{-5}$
	30	$(7.8 \pm 0.1)x10^{-4}$			30	$(11.8 \pm 2.4)x10^{-5}$
	40	$(19.3 \pm 1.2)x10^{-4}$			40	$(40.2 \pm 6.5)x10^{-5}$
	50	$(28.9 \pm 3.5)x10^{-4}$			50	$(79.2 \pm 2.3)x10^{-5}$
	60	$(58.7 \pm 2.8)x10^{-4}$			60	$(213.8 \pm 23.5)x10^{-5}$

Supplementary Table S1. Observed rate constants of the processes **1** → *ent*-**2** and **2** → *ent*-**1** at different pH and temperature values.

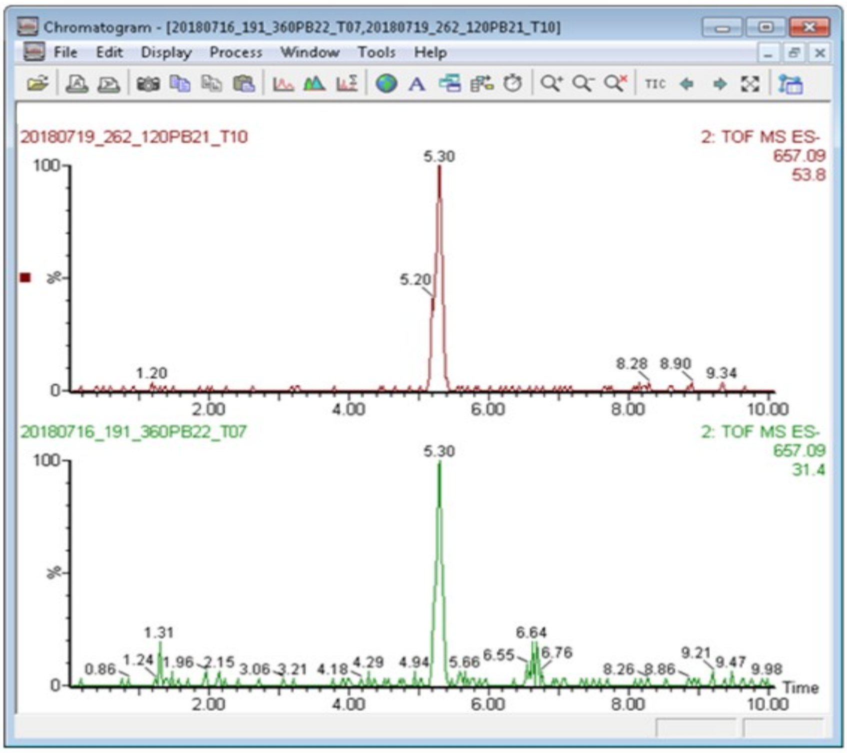

Supplementary Figure S3. LC-MS SIM chromatograms of procyanidin B2 sulfonation product (**7**). The yield of the reaction was low and difficult to draw the kinetic plots (see also Supplementary Figure S4), but these chromatograms proved that the reaction occurred.

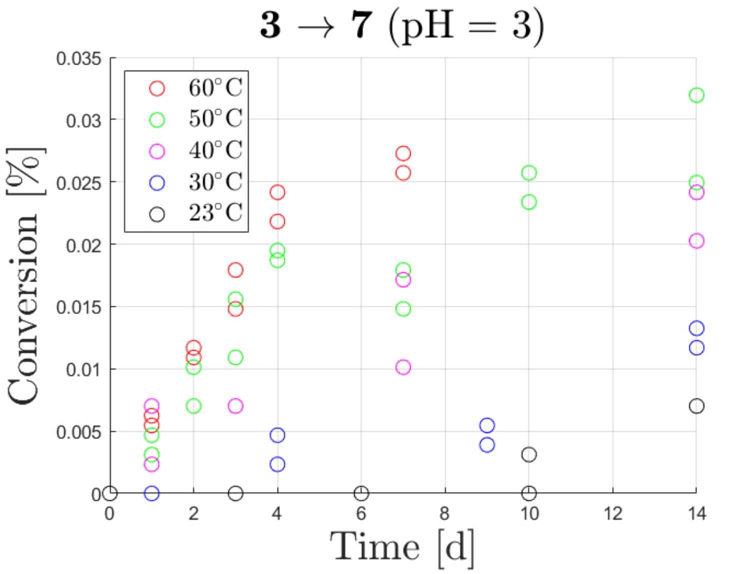

Supplementary Figure S4. Procyanidin B2 sulfonation process (**3** → **7**) at pH = 3 in conversion percentage versus time plot for all tested temperatures. Plot shows negligible amount of product, near the limit of detection of the instrument, moreover not always compatible with increasing formation trend.

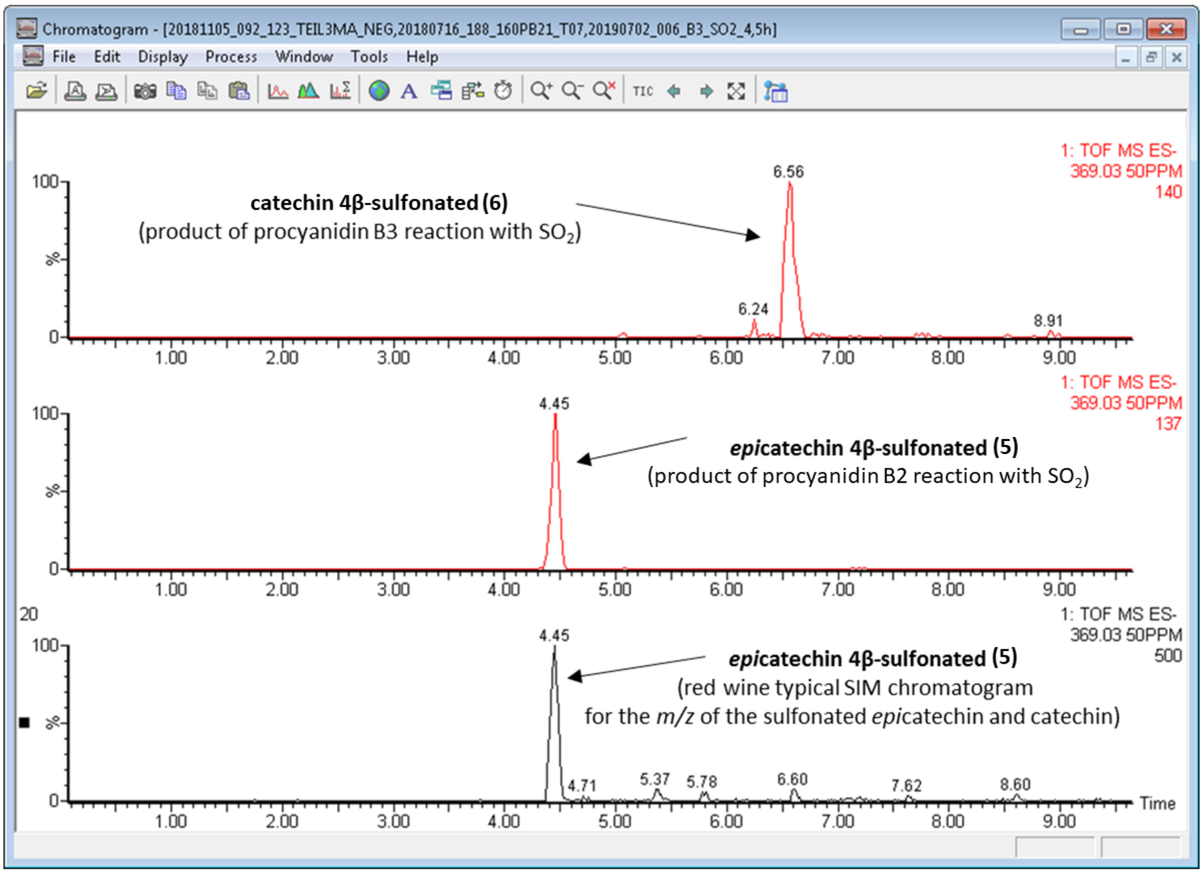

Supplementary Figure S5. The 4β-sulfonated epicatechin and catechin (**5** and **6**, respectively) have the same *m/z* but different retention time, and the major peak for this *m/z* in red wine corresponds to epicatechin 4β-sulfonated.

PROCESS	T [°C]	k_{obs} [d^{-1}]		PROCESS	T [°C]	k_{obs} [d^{-1}]
**3 → 1** pH = 3	23	(1.0 ± 0.1)x10^{-2}		**4 → 2** pH = 3	23	(3.0 ± 0.3)x10^{-3}
	30	(1.8 ± 0.5)x10^{-2}			30	(6.7 ± 0.5)x10^{-3}
	40	(3.4 ± 0.5)x10^{-2}			40	(2.1 ± 0.2)x10^{-2}
	50	(6.1 ± 0.3)x10^{-2}			50	(3.8 ± 0.3)x10^{-2}
	60	(13.7 ± 1.3)x10^{-2}			60	(13.1 ± 1.1)x10^{-2}
**3 → 1** pH = 4	23	(0.7 ± 0.1)x10^{-2}		**4 → 2** pH = 4	23	(1.7 ± 0.1)x10^{-3}
	30	(1.0 ± 0.1)x10^{-2}			30	(5.0 ± 0.5)x10^{-3}
	40	(1.9 ± 0.4)x10^{-2}			40	(1.1 ± 0.2)x10^{-2}
	50	(4.2 ± 0.6)x10^{-2}			50	(3.4 ± 0.4)x10^{-2}
	60	(11.2 ± 2.0)x10^{-2}			60	(9.3 ± 2.3)x10^{-2}

Supplementary Table S2. Observed rate constants of the processes **3** → **1** and **4** → **2** at different pH and temperature values.

PROCESS	T [°C]	R^2 []	
		pH = 3	pH = 4
**1 → 5**	23	0.977	0.966
	30	0.987	0.989
	40	0.998	0.967
	50	0.997	0.996
	60	0.984	0.994
**2 → 6**	23	0.991	0.967
	30	0.995	0.985
	40	0.992	0.988
	50	0.996	0.999
	60	0.999	0.997
**3 → 5**	23	0.981	0.985
	30	0.988	0.972
	40	0.995	0.986
	50	0.988	0.988
	60	0.976	0.953
**4 → 6**	23	0.951	0.999
	30	0.990	0.996
	40	0.956	0.981
	50	0.979	0.958
	60	0.982	0.971

Supplementary Table S3. R^2 for all data fitting in Fig. 4 and Fig. 7 (sulfonation process starting from epicatechin (**1**), catechin (**2**), procyanidin B2 (**3**) and B3 (**4**)).
